# Description of two *Serratia marcescens* associated mastitis outbreaks in Finnish dairy farms and a review of literature

**DOI:** 10.1186/s13028-019-0488-7

**Published:** 2019-11-14

**Authors:** Mari Johanna Friman, Marjut Hannele Eklund, Anna Helena Pitkälä, Päivi Johanna Rajala-Schultz, Merja Hilma Johanna Rantala

**Affiliations:** 1 Department of Veterinary Production Animal Medicine, Faculty of Veterinary Medicine, University of Helsinki, Paroninkuja 20, 04920 Saarentaus, Mäntsälä, Finland; 2 Department of Equine and Small Animal Medicine, Faculty of Veterinary Medicine, University of Helsinki, PO Box 57, 00014 Helsinki, Finland

**Keywords:** Bovine mastitis disinfectant, Outbreak, *Serratia marcescens*, Teat dip

## Abstract

**Background:**

Infection with *Serratia* spp. have been associated with mastitis outbreaks in dairy cattle herds. Environmental contamination or a point source, like a teat dip product, have often been observed to be potential sources of such outbreaks. We describe two *Serratia marcescens* associated mastitis outbreaks associated with a contaminated teat dip containing a tertiary alkyl amine, *n*,*n*-bis (3-aminopropyl) dodecylamine in two dairy cattle farms in Finland. *S. marcescens* strains isolated from milk and environmental samples were identified by the MALDI-TOF method.

**Results:**

Six specimens (n = 19) on Herd 1 and all specimens (n = 9) on Herd 2 were positive for *S. marcescens*. Positive specimens were from mastitis milk and teat dip liquid and equipment. Bacteria were not isolated from the unopened teat dip canister. The same clone of *S. marcescens* was isolated from milk samples and teat dip samples within the farms. Pulsed field gel electrophoresis results to the *S. marcescens* isolates from these two different herds were tested with unweighted pair-group method using arithmetic average clustering analysis. The isolates were not same clone in both herds, because similarity in that test was only 75% when cut-off value to similarity is 85%.

**Conclusions:**

Our investigation showed that the post milking teat dip and/or temporary containers were contaminated with *S. marcescens* and these were most likely the sources for new mastitis cases. The negative result from the unopened teat dip canister and positive results from refillable containers demonstrated that the product itself was not contaminated with *S. marcescens* at the production unit, but became contaminated at the farm level.

## Background

*Serratia* species are rod-shaped Gram-negative bacteria, which were recently classified under the new family of the order *Enterobacterales: Yersiniaceae* [1]. To date, 20 different *Serratia* spp. have been described [2]. *Serratia* spp., in particular *Serratia marcescens*, and *Serratia liquefaciens* are ubiquitous environmental bacteria capable of causing opportunistic infections in humans and many animal species [3] including mastitis in dairy cows [4]. These organisms have been isolated from water, soil, different types of plants and insects [5], farm environment like bedding [6] and milking parlor [7] and from feces of dairy cows [3, 7]. *Serratia* spp. have also been detected in 1.3–2% of bulk tank milk samples [8, 9]. *Serratia* spp. can form biofilm on inanimate surfaces [3] and produce heat resistant enzymes, thus they are capable of causing spoilage at different points of milk processing [10, 11]. However, certain subspecies like *S. liquefaciens* can also be beneficial by contributing to ripening of raw milk cheese products due to their proteolytic activity [12].

*Serratia marcescens* and *S. liquefaciens* have been associated with mastitis outbreaks in dairy cattle herds [6, 13, 14]. Environmental contamination, or a point source that harbours the bacterium, has often been observed to be a potential source of the outbreaks [15, 16], similar to outbreaks in human hospitals [17]. We describe two *S. marcescens* mastitis outbreaks associated with a contaminated teat dip in two dairy farms in Finland. In addition, the literature about *Serratia* spp. mastitis outbreaks in dairy cows is reviewed.

## Methods

### Farms and data collection

Owners of two dairy farms, located over 300 km apart, contacted the Production Animal Hospital of the Faculty of Veterinary Medicine, University of Helsinki, in 2016 due to several *Serratia* mastitis cases that had occurred on the farms during the past years. The first case of *S. marcescens* from mastitis milk sample in Herd 1 was in August 2011, and in Herd 2 in September 2016. After the farmer contacts, an outbreak investigation was initiated to find a possible source of the organism and to control the outbreaks.

Information on farm type, herd size, annual milk yield, milking and hygienic practices, including the use of disinfectants, and laboratory reports of tested mastitis milk samples (from 2014 to 2016) were obtained from the farmers. Milk samples had been tested in a milk processing company’s laboratory (Valio Ltd, Lapinlahti, Finland) using multiplex real-time PCR method (PathoProof™ Mastitis Complete-16 Kit, A Thermo Scientific™, Vantaa, Finland). Data about udder health in the herds were retrieved from the national health care recording system, ETT Naseva [18], per the owners’ permission.

### Sample collection

During the farm visit (08/2016) to Herd 1 to investigate the outbreak, all lactating cows (n = 33) were tested with the California Mastitis Test (CMT). Quarter milk samples for bacteriological culture were collected by the investigating veterinarian from all quarters that showed CMT score > 2 (n = 5) on a 1–5 scale [19]. Milk samples were collected into 10 mL plastic tubes (Linkoputki 16 × 100 mm Plastone, Mekalasi, Helsinki, Finland). One of the five samples was from an acute mastitis case and the rest were from subclinical mastitis. In addition, one frozen quarter milk sample from the most recent acute mastitis case that had occurred within a week prior to the herd visit was available for testing. Thus, six mastitis milk samples were cultured from Herd 1.

To find the potential source of *S. marcescens* in the environment, surface samples for bacteriological culture were taken using sterile cotton swabs (M40, Transystem Amies Agar Gel without charcoal, Copan Diagnostics, CA, USA) from a water faucet (n = 1), milking unit liners (n = 2), washing centres of the milking units (n = 2), water cups of the cows (approximately 5 × 5 cm area) (n = 1) and from the nozzle of the teat dip spray bottle (n = 1). Also, 5 mL samples of water from the bucket (n = 2), where udder towels were kept during milking, and from the teat dip product (n = 3) were collected into the same kind of sterile plastic tubes as milk samples. The specimens from the teat dip product were collected by spraying the teat dip product from a refillable spray bottle, by pumping it through a reusable plastic pump that was attached to the original opened canister, as well as by taking the product straight from an opened canister. In addition, five litres of drill well water was collected into a sterile can for bacteriological investigation. In total, the number of environmental samples was 13.

Samples (n = 9) from Herd 2 were collected by the farmer. These consisted of quarter milk samples from two cows, each with one clinical mastitis quarter, and seven teat dip samples. The teat dip product from an opened teat dip canister (n = 1), and all teat dip spray bottles (n = 6) were sampled as described for Herd 1.

In addition, a new, unopened 20 L canister of the teat dip product (Viri-Dip Plus, Oy Teollisuushankinta TH Ab, Kokkola, Finland) was ordered straight from the importer of the product to our laboratory to test the bacterial quality of the product.

### Microbiological methods

#### Culturing and identification

All samples were cultured immediately after arrival to the Laboratory of Production Animal Hospital (University of Helsinki, Mäntsälä, Finland). Culture swabs and 10 µL of fluid samples (milk, teat dip and bucket water) were streaked onto 5% sheep blood agar plates (Tammer-Tutkan Maljat Oy, Tampere, Finland), and incubated in ambient air at 37 °C for up to 48 h. Preliminary identification of the species was made according to the guidelines of NMC [20].

Well water specimen (5 L) was filtered through a commercial membrane filter with a pore size of 0.45 µm (Millipore Corporation, MA, USA), after which the filter was placed aseptically on a nonselective chromogenic agar (UriSelect, Bio-rad Finland Oy, Helsinki, Finland), and incubated at 35 °C for up to 48 h.

Identification of the isolates, that were tentatively identified as *Serratia* spp. based on colony and bacterium morphology, were further confirmed in the Clinical Microbiology Laboratory of the Faculty of Veterinary Medicine (University of Helsinki, Finland), by matrix-assisted laser desorption ionization-time of flight mass spectrometry using MALDI Biotyper Microflex LT (Bruker Daltonics GmbH, Bremen, Germany) and MALDI Biotype MSP Identification Standard Method 1.1.; using score ≥ 2.00 for species level identification. In addition, 16S rRNA gene sequence based identification was performed for seven isolates. Bacterial supernatant in InstaGene Matrix (Bio-Rad Laboratories Inc., CA, USA) was used as a DNA template. The 16S rRNA gene was amplified with 0.25 µM of universal primers F8 5′-AGAGTTTGATCCTGGCTCAG-3′ and R1541–1522 5′-AAGGAGGTGATCCAGCCGCA-3′ [21]. The amplicons were sequenced with primers F19–38 5′-CTGGCTCAGGAYGAACGCTG-3′ [22], R519 5′-GTATTACCGCGGCTGCTG-3′ [23], F926 5′-AACTCAAAGGAATTGACGG-3′ [24], and with R1541–1522. The trimmed sequences of 1500 base pairs were analysed by CLC Main Workbench Software (version 8.0, Qiagen, Denmark) and compared to the BLAST/NCBI database [25] for bacterial identification.

The susceptibility testing was done with a disk diffusion method [26, 27] for the following antimicrobials: amikacin, cefpodoxime, enrofloxacin, gentamicin, meropenem and sulfamethoxazole/trimethoprim (Oxoid Ltd, Hampshire, UK). Of these cefpodoxime, amikacin and meropenem were tested only for resistance surveillance purposes. Production of ESBL and AmpC enzymes was investigated with ESBL and AmC Detection Disk Set together with ESBL/AmpC Calculator (Mast Group Ltd, Merseyside UK).

#### Genotyping of *S. marcescens* strains

Fresh overnight cultures were used to prepare agar blocks for pulsed field gel electrophoresis (PFGE). Bacterial mass was suspended into cold 100 mM EDTA to achieve density of 8.5 McFarland units (Den-1B McFarland Densitometer, Grant-bio, Grant Instruments Ltd., Cambridgeshire, UK), followed by heating in +75 °C for 10 min [28]. After this the PulseNet *Escherichia coli* O157 PFGE protocol [29] with digestion of XbaI enzyme (New England Biolabs Inc., MA, USA) was followed. Separation of DNA fragments was done by using Chef DR III system (Bio-Rad Laboratories Inc., CA, USA). The fragments were visualized by SYBR Safe DNA staining (Thermo Fisher Scientific, Waltham, MA, USA) and imaged with AlphaImager HP (Alpha Innotech, Genetic Technologies Inc., FL, USA). PFGE patterns were examined using GelComparII software (version 6.6 Applied Maths NV, Belgium) to perform UPGMA (unweighted pair-group method using arithmetic average clustering) based analysis with the Dice similarity coefficient. Similarity cut-off was 85% to separate clusters and optimization and position tolerance were both set at 1.5%.

### Literature review

Literature search concerning *Serratia* spp. in dairy cows was performed by utilizing Pubmed (https://www.ncbi.nlm.nih.gov/pubmed/) and Web of Science (https://webofknowledge.com/) databases. Keywords used for the search were ‘cow’ AND ‘mastitis’ AND ‘Serratia’ AND as subject-specific terms. All article types were included and reference lists in these were scanned to identify additional references that were not found in electronic database search.

## Results

Background information on both herds is summarised in Table 1. Both farms were tie stall herds with less than 50 cows (Ayrshire, Herd 1; Holstein–Friesian, Herd 2) with average milk production > 10,000 L/year/cow. The herds did not have any apparent problems in their hygienic practices or in the farm environment. In both herds, quarter milk samples for bacteriological testing were taken routinely if clinical signs of mastitis were observed or if elevated cell count of the milk was detected.

**Table 1:** Characteristics of two Finnish dairy farms with high incidence of *Serratia marcescens* cases

Character	Herd 1	Herd 2
Farm type	Tie stall, 5-year old barn	Tie stall, renovated 15 years ago
Herd size	45	37
Cow breed(s)	Ayrshire	Holstein–Friesian
Annual average milk yield (litre/cow)	11,111	10,058
Stalls	Rubber mattresses, no bedding	Saw dust bedding
Environment	Clean environment and good hygiene practices. Cows at pasture in summertime. Pasture clean and dry, but a muddy area around the feeding rack and water tank	Clean environment and good hygienic practices except that the stalls for the biggest cows were too short and thus dirty. Cows at pasture during summertime
Milking devices	Six milking units with automatic take offs	Six milking units with automatic take offs
Udder and teat cleaning before milking	Separate moist towels for each cow, machine washing and drying of towels between milkings	Separate moist towels for each cow, machine washing and drying of towels between milkings
CMT test	Routinely on all cows once a week and always if the cow has signs of mastitis	On all cows if bulk tank milk SCC elevated, and for cows with clinical signs of mastitis
Bacteriological testing of milk samples (PCR)	If CMT > 2	If CMT > 2
Post milking disinfection	Viri-Dip plus^a^, re-usable spray bottles that were filled daily from a storage canister and rinsed with tap water between the fillings. Storage canister had a pump that was moved to a new canister without cleaning	Viri-Dip plus^a^, re-usable spray bottles that were filled daily from a storage canister and rinsed with tap water between the fillings. Storage canister had a pump that was moved to a new canister without cleaning
Other practices	Selective dry cow therapy, approximately 50% of cows treated. Udder supporters were used for almost all cows	Selective dry cow therapy, only for few cows. Udder supporters lined with newspapers were used for cows with large udders.
Water quality	Well water met the legislative requirements	Well water met the legislative requirements
Most commonly used antimicrobial for treating mastitis	Procaine penicillin	Procaine penicillin

^a^ A product containing *n*,*n*-bis (3-aminopropyl) dodecylamine, lactic acid, allantoin, glycerol and sorbitol (Viri-Dip plus, Novadan ApS, Danmark)

**Fig. 1:**
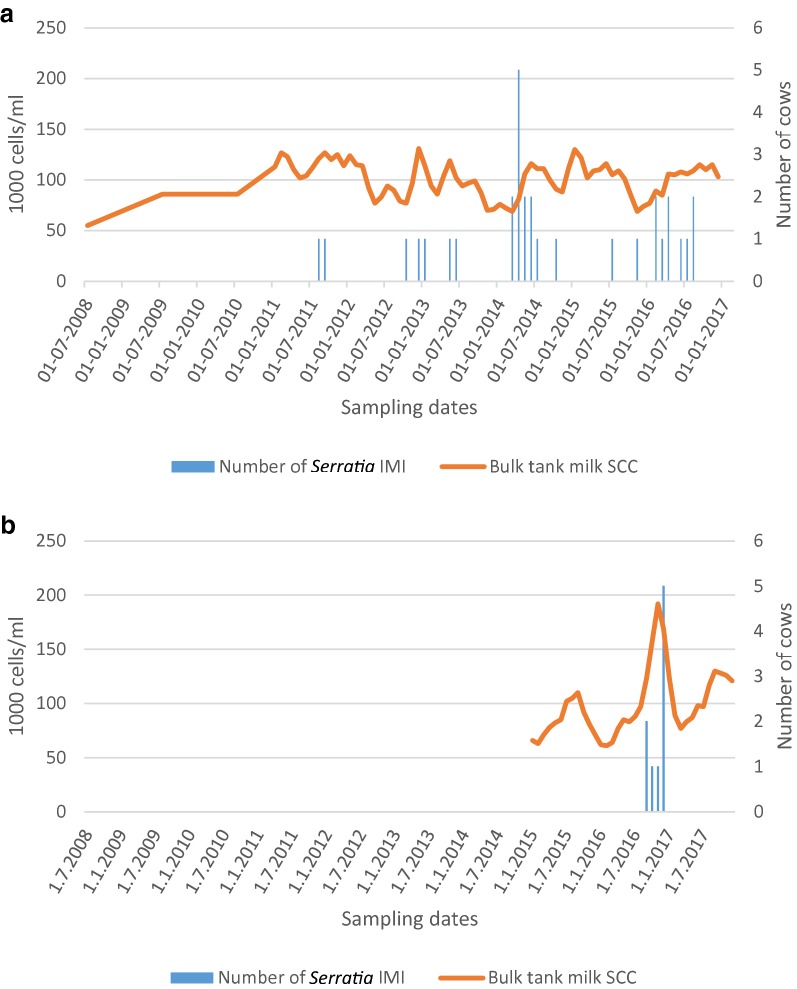
**a** Number of *Serratia marcescens* intra-mammary infections (IMI) and somatic cell count (SCC) in bulk milk of Herd 1. **b** Number of *Serratia marcescens* IMI and SCC in bulk milk of Herd 2

In Herd 1, since the first case of *S. marcescens* in August 2011 *S. marcescens* DNA had been isolated from 35 milk samples (8/2011–8/2016). Of the milk samples analysed during the period of 2014–2016, 71% (90/126) were positive for pathogens included in the PCR kit used in the testing laboratory. DNA of *S. marcescens* was observed in 39% of the positive samples (35/90). Other bacteria detected were non-aureus staphylococci (NAS, n = 33/90, 37%), *Staphylococcus aureus* (n = 10/90, 11%), *E. coli* (n = 6/90, 7%) and miscellaneous bacterial species (n = 6/90, 7%). *S. marcescens* mastitis cases and somatic cell count (SCC) in bulk milk are shown on the timeline in Fig. 1a. The number of treated mastitis cases in the herd varied from 5 to 28/100 cows/year during years in 2012–2016, being the highest a year after the first *S. marcescens* mastitis case. A marked proportion of all cows (23%) was slaughtered during the year 2012 because of *S. marcescens* mastitis. No antimicrobial treatments had been given to *S. marcescens* infected cows.

In Herd 2, the first case of *S. marcescens* mastitis was observed in September 2016. During the period of 2015–2017, 43 mastitis milk samples had been analysed with the PCR-method, of which 26 (60%) yielded positive results. DNA of *S. marcescens* was detected in nine samples (n = 9/26, 35%), followed by NAS (n = 9/26, 35%), *S. aureus* (n = 2/26, 8%), *Streptococcus dysgalactiae* (n = 2/26, 8%) and other species (n = 4/26, 15%). All *S. marcescens* positive milk samples had been collected between September 2016 and December 2016. *Serratia marcescens* mastitis cases and SCC in bulk milk are shown on the timeline in Fig. 1b. Incidence of mastitis cases increased from 5/100 cows/year in 2015 to 29/100 cows/year in 2016 and the proportion of cows slaughtered due to mastitis among all slaughtered cows increased from 8% in year 2015 to 13% in year 2016.

### Bacteriological results to detect potential source

**Fig. 2:**
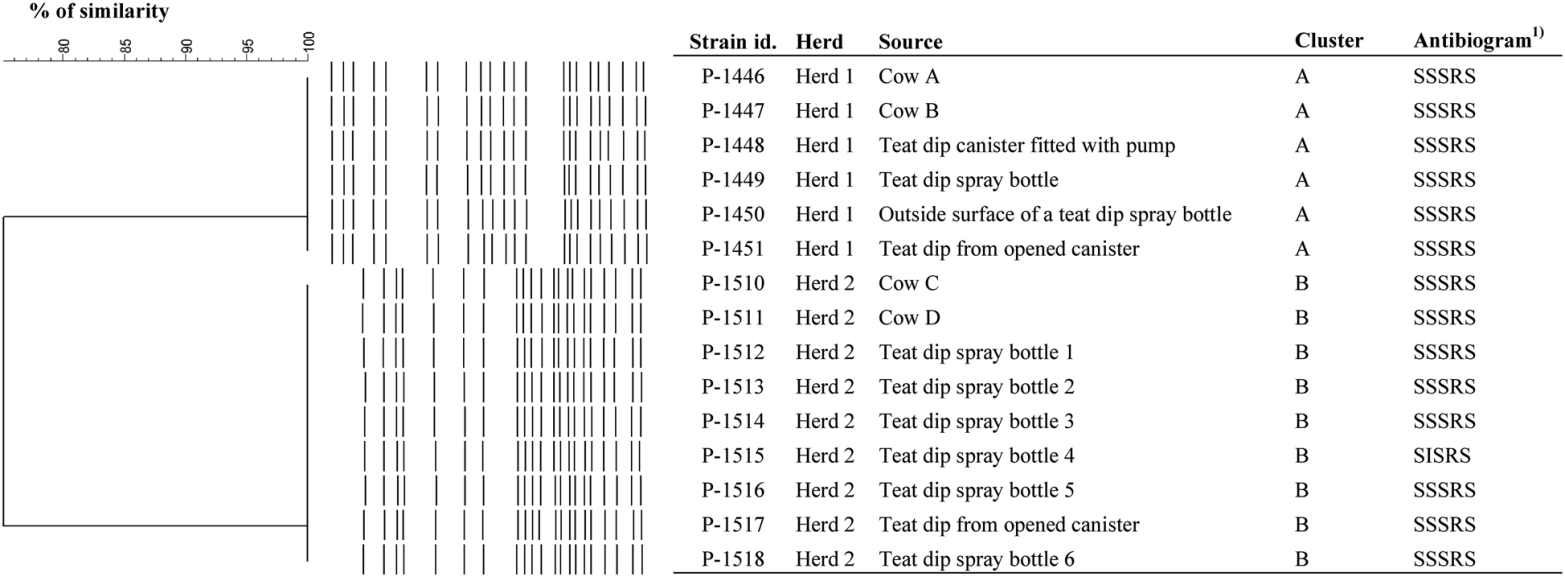
PFGE and antimicrobial susceptibility patterns of the *Serratia marcescens* isolates from the two herds. ^1)^Antibiogram is represented in the following order: sulfamethoxazole/trimethoprim, enrofloxacin, gentamicin, cefpodoksime and amikacin

Regarding Herd 1, six specimens out of 19 yielded *S. marcescens* growth: two mastitis milk samples and all four teat dip/teat dip container samples (fluid from an opened canister, fluid pumped from the canister, fluid from the spray bottle and the swab sample taken the nozzle of the spray bottle). Other milk samples, unopened teat dip canister, towel bucket water, drill water, water cup, liner or faucet samples were negative for *S. marcescens* although heavy mixed growth was cultured from the water cup and liner samples. All six *S. marcescens* isolates from Herd 1 were identical in the PFGE analysis. PFGE patterns and antimicrobial resistance profiles of the isolates are represented in Fig. 2. Three isolates indicated AmpC activity.

Of Herd 2 samples, all nine specimens (two clinical mastitis samples, teat dip from the opened canister, and six teat dip specimens from different spray bottles) were positive for *S. marcescens*. All nine isolates of Herd 2 were identical with similar antibiogram and PFGE profile (Fig. 2). All isolates expressed AmpC activity.

With this epidemiological “golden standard genotyping method”, the *S. marcescens* strains between the two farms showed 75% difference in the UPGMA based cluster analysis (Fig. 2), thus indicating epidemiological difference of the strains, similarity cut-off being 85%.

Table 2 summarizes the 15 case reports that were found in the literature review. Beside the case reports found, additional 16 articles dealt with pathology, immunology, antibiotic treatment and resistance, prevalence of *Serratia* spp. in bulk milk or mastitis milk samples. In many *Serratia* mastitis outbreaks the source of *Serratia* spp. remained unresolved (n = 7/15). The teat disinfectant or equipment used in teat-dipping were shown to be the source for *Serratia* mastitis outbreak in four cases (n = 4/15) and suspected to be the source in one case. Only Lium [30] reported a fatal *Serratia* associated mastitis case, whereas subclinical and chronic mastitis were more commonly reported. The reviewed literature suggested that culling was more common among *Serratia* infected cows than in a healthy population.

**Table 2:** Literature review of *Serratia* spp. associated mastitis outbreaks

Published [references]	Country	Animals/farm type	Suspected source and predisposing factors	Proportion of infected animals	Culling
*Serratia marcescens*					
[4]	USA	50 herds in multiple states	Chlorhexidine-containing teat disinfectant, detected *S. marcescens* in open containers and dip-cups. The product contamination appeared to occur on the farm and resulted in a within-herd outbreak of a farm-specific *S. marcescens* strain	N	Culling was more common among *Serratia*-infected cows than control cows
[50]	Greece	156 milking ewes	Teat dip cup (not observed from teat dip: a quaternary ammonium base)	Clinical, incidence 5%, prevalence 16%	0
[7]	USA	1 herd; 240–260 Holstein cows, open lots	The dirt pack of lots (n = 3 positive sample) and the milking parlor floor (n = 2 positive samples)	8–17% of tested composite milk samples positive	11 cows
[6]	USA	1 herd; 1000 Holstein cows, manure-straw paddocks	Isolated from lactating cows’ bedding samples and one cup liner sample	13–18% of tested composite milk samples positive	N
[34]	USA	1 herd; 190 Jersey cows, stall stanchion barn	Suspected chlorhexidine digluconate 0.5% post milking teat dip	21 cows	4 cows
[51]	Canada	1 herd; 85 cows	Indefinite Predisposing factors: overmilking and wet udders	Subclinical IMI c. 10 cows, together with *Pseudomonas aeruginosa* mastitis	N
[13]	Sverige	1 herd; 37 cows, tie-stall farm	Not investigated	Almost all cows infected	15 cows
[15]	USA	45 Holstein cows, straw bedded stalls	A quaternary complex teat dip	6 cows during 2 months	N
[30]	Norway	University hospital, 1 cow	Not investigated	Acute mastitis	Died
[33]	Great Britain	6 herds	Indefinite Predisposing factors: a faulty water supply (i.e. ponds, springs, wells, brooks and flooding)	N	N
[40]	Canada	1 herd; 24 cows	Indefinite	11 cows	N
*Serratia liquefaciens*					
[14]	USA	1 herd; 41 Holstein cows, loafing barn, straw bedding	Indefinite Predisposing factor: frostbite in teats	30 IMI cases, *S. liquefaciens* bacteria isolated from 11 IMI cases, bacteriologic testing not performed n = 19	N
[41]	Australia	1 herd; 120 Friesian cows, pasture	Indefinite	5 clinical IMI, 1 subclinical IMI in mid-lactation period during 3 weeks period	0
*Serratia* spp.					
[52]	USA	1 herd; 120 cows (Jersey and Holstein), 75% free-stall barn, 25% tie-stall barn, recycled manure beddings	Indefinite	75 IMI during 10 years Mainly dry cows (62%)	17% of infected cows
[35]	USA	1 herd; 140 Holstein cows, free-stalls, sand bedding. dry cows: loose housing straw- manure pack	Indefinite Pre/post teat dip 4% hypochlorite group were infected more often than ionophore group Predisposal factor: frost bite and chapping on teat skin	43 cows during 1 year	N

*N* information missing, *IMI* intramammary infection

## Discussion

In both herds the bacterial species in the collected samples was confirmed to be *S. marcescens*. *Serratia marcescens* has been reported to cause both clinical and subclinical mastitis outbreaks during the lactation and the dry period in dairy cows [31]. It is a rare cause for bovine mastitis in Finland [32]. During the outbreak situations described here, however, *S. marcescens* findings increased drastically in both farms. Identical isolates from the cows, the teat dip product and spray bottles indicated transmission of the bacteria from a contaminated point source, the teat dip or spray bottles where the teat dip was temporarily kept, to the cow. Similar to a previous report [4], the negative result from the unopened canister and positive results from temporary containers demonstrated that the product itself was not contaminated with *S. marcescens* at the production unit, but became contaminated at the farm level. Although the original source for *S. marcescens* contamination remained unclear, it is likely that the source of the bacterium was the environment, and that daily practices promoted the contamination of spray bottles and pumps. Amount of the teat dip that was used daily was taken from a storage canister via a pump that was moved to a new canister without cleaning. Also, spray bottles were not machine washed but were only flushed with tap water between the re-fillings. It is not known whether the bottles dried out properly after the washing, prior to re-filling the bottle. Considering the nature of the *S. marcescens* and its ubiquitous presence in the environment, contamination of bottles by water splashes or by contaminated hands is easy in the farm environment. In addition, *Serratia* species are prone to form biofilms. The way the farms handled the teat dip product and its refillable containers was favourable for biofilm formation, which in part could have promoted the existence of *S. marcescens* and its emergence to teats of the cows. However, the presence of biofilms was not evaluated in our study. In this study water appeared not to be a source for *S. marcescens*. On the other hand, relatively few specimens from the water faucet areas or areas where spray bottles were handled were taken and thus this source could have stayed undetected. A faulty water supply has been reported to be predisposing factor to *Serratia* mastitis [33].

Because of the ubiquitous nature of *Serratia* spp. in the environment, these bacteria are abundant on dairy farms. Table 2 contains information on published *Serratia* spp. mastitis outbreaks and sources of bacteria. For example, their presence has been reported from bedding material and floors of a milking parlor [6, 7]. *Serratia* spp. has been isolated from bedding materials in areas where known *Serratia* infected lactating cows are kept, but not from the area of dry cows. Wilson et al. [34] and Hogan and Smith [35] have shown that dairy cows may carry *Serratia* spp. subclinically for several months or even years. Hence it is possible that leaking of milk from Serratia infected cows could further spread the bacteria to the environment. Some cases of Serratia mastitis can cure spontaneously but infected cows can also become chronic carriers. Guardo et al. [36] showed that chronic carriers of *S. marcescens* can be the source of infection on a milking farm. In both farms of this study, several infected cows had been slaughtered rapidly after infection which diminished the risk for cow to cow transmission and possibly prevented the situation from escalating even worse. Udder supporters were used in both farms of this study, which reduced the risk of environmental contamination.

*Serratia marcescens* mastitis is challenging to treat as the organism is intrinsically resistant to many antimicrobials [37, 38] due to multi-drug efflux pump(s) [39]. Mastitis cases caused by *S. marcescens* were not treated with antimicrobials in either of the herds of this study due to a lack of appropriate licensed veterinary products for lactating cows. Intra-mammary liquid neomycin has been used to cure experimentally infected *S. marcescens* quarters [40]. Positive treatment response with intra-mammary neomycin was also documented in a cluster of *S. liquefaciens* mastitis cases in one farm [41]. Acquired resistance is also common in this species although *S. marcescens* isolates of this study were susceptible to tested agents except for cefpodoxime (Fig. 2). Neomycin susceptibility is not routinely tested in our laboratory, which is a limitation of the study. Cefpodoxime resistance is probably explained by the presence of chromosomal AmpC production which is typical for this bacterial species. This was supported by the fact that although the phenotypic ESBL/AmpC testing indicated AmpC activity in majority of the strains, the zone inhibition profiles around the disks were not correspondent to that seen in isolates with plasmid-mediated AmpC production or hyper-production of chromosomal AmpC. However, we did not verify the presence of ESBL/AmpC genes by PCR.

Serratia mastitis outbreaks can be very challenging to control with antiseptics because *Serratia* spp. can carry both chromosomal and plasmid-encoded resistance to certain biocides such as chlorhexidine [42] and quaternary ammonium compounds [39]. *Serratia* spp. has been reported to survive in disinfectant solutions based on quaternary ammonium compounds (QAC), amines and glucoprotamin in reusable disinfection tissue dispensers [43], in chlorhexidine solution [44], and in amphoteric or alkyl amino acetate-based disinfectants [45]. Outbreaks of *Serratia* mastitis in dairy herds have earlier been associated with chlorhexidine-containing teat disinfectants [4] and quaternary ammonium compounds [15]. *S. marcescens* outbreaks described in this study are the first ones associated with a teat dip containing a tertiary alkyl amine, *n*,*n*-bis (3-aminopropyl) dodecylamine (CAS number 2372-82-9; known also as laurylamine dipropylenediamine and several other acronyms), as an active substance. The substance is used as a surfactant, disinfectant, biocide as well as preservative in cosmetic products [46]. In the teat disinfectant product, the concentration of the active substance is 0.4% corresponding 4000 ppm. *N*,*n*-bis (3-aminopropyl) dodecylamine is reported to be active against vegetative forms of bacteria, but presence of proteins or organic material can reduce its activity [47]. Efficacy testing results were not available for the teat dip product used in these farms, but the active substance is currently under review for a use as a biocide according to EU Biocidal Products Regulation (EU 528/2012). We were able to find one publication in which the effect of the similar product containing 0.5% of the same active substance (CAS 2372-82-9, aminopropyl lauramine) was compared with iodine-based disinfectant [48]. It appeared that 0.5% CAS 2372-82-9 containing product was far less effective in reducing bacterial colony counts on the teats compared to 0.2% iodine-based disinfectants [48]. This might indicate that the teat disinfectant based to CAS 2372-82-9 is not effective enough for this purpose. Like other Gram-negative bacteria, *Serratia* spp. may also develop higher resistance if they are exposed to sub-inhibitory concentrations of tenside-based disinfectants, QAC or chlorhexidine because of irreversible changes in the cell wall, or if they are growing sheltered by biofilm [49]. In our study we did not have resources to test disinfectant resistance in *S. marcescens* strains.

Proportion of infected cows in our herds were slightly higher than those in published outbreaks, where approximately a tenth of herds’ cows became infected during the outbreak. An exception is the outbreak reported by Isaksson and Holmberg [13], where incidence and mortality were high. Culling of the cows due to *Serratia* mastitis caused high losses for the farmers of our herds. Increased bulk tank milk cells have been reported by Wilson [34] and Bowman [14] and we found parallel change in our cases; bulk tank milk cells (Fig. 1b) increased clearly in Herd 2 at the same time as the first *Serratia* mastitis case was detected, but the change was not so obvious in Herd 1 (Fig. 1a).

The farmers were instructed to destroy all old teat-dip utensils and change the post-dipping product to other product, which contain another active substance. The farmers were advised to use blanket dry cow therapy with product containing neomycin for a period of 1 year. No new *Serratia* mastitis cases were diagnosed during the following year after those changes were implemented in the herds.

## Conclusions

*Serratia marcescens* was isolated repeatedly from quarter milk samples from lactating cows in both farms. Our investigation showed that the post milking teat dip and/or refillable containers were contaminated with *S. marcescens* and these were most likely the sources for new cases. The same clone of *S. marcescens* was isolated from milk samples and teat dip samples within the farms suggesting the same source.

The plastic pump was moved by the farmers from one container to another without cleaning, and this could have maintained the infection. The unopened teat disinfectant container was not found to be contaminated, which suggested that the teat dip bottles or pump mechanism probably became contaminated on the farms. The *S. marcescens* most likely exists in the farm environment as the bacterium is common.

After changing the teat-dip product and related equipments herds have been free of new *Serratia* mastitis cases.

## Data Availability

The datasets used and/or analyzed during the current study are available from the corresponding author on reasonable request
